# A program to compute the soft Robinson–Foulds distance between phylogenetic networks

**DOI:** 10.1186/s12864-017-3500-5

**Published:** 2017-03-14

**Authors:** Bingxin Lu, Louxin Zhang, Hon Wai Leong

**Affiliations:** 1 Department of Computer Science, National University of Singapore, 13 Computing Drive, Singapore, 117417 Singapore; 2 Department of Mathematics, National University of Singapore, 10 Lower Kent Ridge, Singapore, 119076 Singapore

**Keywords:** Phylogenetic network, Cluster containment problem, Tree containment problem, (Soft) Robinson–Foulds distance, Exponential-time algorithm

## Abstract

**Background:**

Over the past two decades, phylogenetic networks have been studied to model reticulate evolutionary events. The relationships among phylogenetic networks, phylogenetic trees and clusters serve as the basis for reconstruction and comparison of phylogenetic networks. To understand these relationships, two problems are raised: the tree containment problem, which asks whether a phylogenetic tree is displayed in a phylogenetic network, and the cluster containment problem, which asks whether a cluster is represented at a node in a phylogenetic network. Both the problems are NP-complete.

**Results:**

A fast exponential-time algorithm for the cluster containment problem on arbitrary networks is developed and implemented in C. The resulting program is further extended into a computer program for fast computation of the Soft Robinson–Foulds distance between phylogenetic networks.

**Conclusions:**

Two computer programs are developed for facilitating reconstruction and validation of phylogenetic network models in evolutionary and comparative genomics. Our simulation tests indicated that they are fast enough for use in practice. Additionally, the distribution of the Soft Robinson–Foulds distance between phylogenetic networks is demonstrated to be unlikely normal by our simulation data.

**Electronic supplementary material:**

The online version of this article (doi:10.1186/s12864-017-3500-5) contains supplementary material, which is available to authorized users.

## Background

Since Darwin’s *The Origin of Species*, the evolutionary history of life has been widely depicted as phylogenetic trees. However, the simplified tree-like evolutionary models are being challenged by the accumulating amount of evidence of lateral genetic transfer between lineages, particularly in prokaryotes [1–3]. Additionally, other reticulate evolutionary events also cause complications in constructing tree-like models, such as hybridization and introgression between species [4, 5], and recombination of various forms [6]. The recognized limitations of phylogenetic trees motivated the adoption of phylogenetic networks to model these reticulation events [7, 8]. Phylogenetic networks can be used to either visualize conflicting phylogenetic information or model reticulation events explicitly. The former are typically unrooted, whereas the latter are rooted, which is the focus of this study. In recent years, phylogenetic networks have been the subject of intensive theoretic studies [9–12]. However, considerable challenges in reconstructing phylogenetic networks still exist [13].

In a phylogenetic tree, the taxa below a node form a unique subset of the taxa, called its cluster. A phylogenetic tree is uniquely determined by the set of “nested" clusters in the tree (see, for example, [10]).

A phylogenetic network is a generalization of a phylogenetic tree in which there are additional reticulation nodes, which are the nodes with an in-degree of at least two. Since most gene families have tree-like evolutionary histories, the network model of the evolution of a set of genomes is often built and validated by checking its consistency with the available related gene trees and/or clusters [10].

In a phylogenetic network, a non-reticulation node is called a tree node. Each tree node represents a cluster and a set of soft clusters. Similar to the case of phylogenetic tree, a node’s cluster consists of all taxa below it, whereas its soft clusters are the clusters represented by this node in the phylogenetic trees that are displayed in the network. Here, a phylogenetic tree is said to be displayed in a phylogenetic network if it can be obtained by deleting all but one incoming edges from each reticulation node and then contracting all the nodes of degree two.

The tree containment problem (TCP) and the cluster containment problem (CCP) have arisen from reconstructions of phylogenetic networks [14]. The TCP asks whether a phylogenetic tree is displayed in a phylogenetic network. The CCP asks whether a cluster is a soft cluster of some tree node in a phylogenetic network. Both the TCP and CCP are NP-complete [10, 14], even for restricted networks [15].

A polynomial-time algorithm for the CCP is given for reticulation-visible networks [10]. A network is reticulation-visible if, for each reticulation node, a leaf exists such that every path from the network root to the leaf contains this reticulation node. Recently, a linear-time algorithm is presented for the CCP on this class of networks [16]. Given that a large fraction of phylogenetic networks are not reticulation-visible [17], however, it is necessary to develop an algorithm for the CCP for arbitrary networks for the following reason.

Measuring the dissimilarity between phylogenetic networks is important for assessing a network reconstruction method. One of the metric functions that has been proposed for this purpose is the Robinson–Foulds (RF) distance, which is a generalization of the same metric for phylogenetic trees. Simply put, it is the half of the cardinality of the symmetric difference of the two sets of clusters respectively contained in the two networks [18]. It takes linear-time to compute the RF distance between phylogenetic networks [10].

By replacing clusters with soft clusters, we obtain the Soft Robinson–Foulds (SRF) distance [10]. Since the CCP is NP-complete, there is unlikely a polynomial-time algorithm for computing the SRF distance. To the best knowledge of the authors, only a straightforward method has been implemented in the software Dendroscope [19]. The method exhaustively searches the clusters that are in a phylogenetic tree displayed in one network but are not in any phylogenetic tree displayed in another.

Recently, Gunawan et al. [20] developed a computer program for solving the TCP on arbitrary networks. Although it has exponential-time complexity in the worst case, it runs fast enough to be used in practice.

Here, we first develop an algorithm for the CCP by using the decomposition theorem in [16]. We then extend it into an algorithm for computing the SRF distance. We implemented these two algorithms in C and tested them on empirical and simulated network datasets. As an application of the programs, we examined the differences of networks reconstructed for two datasets in the literature. We also conducted a preliminary study of the distributions of the RF and SRF distances in the phylogenetic network space.

## Methods

We first introduce the basic concepts and notation, then recap the decomposition technique for arbitrary phylogenetic networks, and finally describe the algorithms for the CCP and the SRF distance.

### Concepts and notation

Let *X* be a set of taxa. A rooted phylogenetic network (network for short) over *X* is an acyclic digraph in which the leaves (i.e., nodes of out-degree zero) are bijectively mapped to *X*. A taxon typically represents some extant organism or species. A network has a unique root (of in-degree zero).

There can be two types of internal nodes in a network: *tree nodes*, which include the root and nodes of in-degree one and out-degree of at least one, and *reticulation nodes*, which have out-degree one and in-degree of at least two. The tree nodes represent speciation events and the reticulation nodes represent reticulation events. We allow degree-two nodes in a network.

Here, we use the following notation for a network *N*:

- *T*(*N*): the set of tree nodes in *N*.
- *L*(*N*): the set of leaves in *N*.
- *R*(*N*): the set of reticulation nodes in *N*.
- *V*(*N*): the set of all nodes in *N*, namely *T*(*N*)∪*L*(*N*)∪*R*(*N*).
- *E*(*N*): the set of edges in *N*.
- *ρ*(*N*): the root of *N*.
- *N*−*E*: the subnetwork (*V*(*N*),*E*(*N*)∖*E*) for a subset *E*⊆*E*(*N*).
- *N*−*S*: the subnetwork (*V*(*N*)∖*V*(*S*),*E* ^′^), where *E* ^′^={(*x*,*y*)∈*E*(*N*) | {*x*,*y*}⊆*V*(*N*)∖*V*(*S*)} for a subnetwork *S* of *N*.

For *u*,*v*∈*V*(*N*), *u* is a *parent* of *v* and *v* is a *child* of *u* if (*u*,*v*)∈*E*(*N*). We use *c*(*r*) to denote the unique child of *r*∈*R*(*N*). If there is a direct path from *u* to *v*, *v* is called a *descendant* of *u*.

We use [ *r*]_*N*_ to denote the subnetwork below *r*∈*V*(*N*), which consists of all the descendants of *r* and the edges between them in *N*. For a leaf *ℓ* below *r*, we use *N*−[ *r*]_*N*_+*ℓ* to denote the subnetwork obtained by replacing [ *r*]_*N*_ with *ℓ* so that *ℓ* becomes the child of *r*.

If each reticulation node in a network has exactly two parents, the network is *bi-combining*. A bi-combining network is *binary* if each tree node is of out-degree two. A phylogenetic tree is a binary network without reticulation nodes. If the unique child of each reticulation node in a network is a tree node or a leaf, this network is called *reduced*.

Following Gunawan et al. [16], we allow a network to have dummy nodes (i.e., unlabelled nodes of out-degree zero) because such a network may be generated in a recursive step of our algorithms.

Given the set of taxa *X*, a *cluster* is any proper subset of *X* (excluding the empty set and the full set). A cluster is *trivial* if it contains only one element.

In a phylogenetic tree *T* over *X*, each non-root node induces a unique set of taxa that consists of the labels of the leaves below the node, which is called the cluster of the node. A cluster is in *T* if it is the cluster of some node in *T*.

Given a network *N* over *X* and a phylogenetic tree *T* over *X*, we say that *T* is *displayed* in *N* if *T* can be obtained from *N* by the following operations: removing all but one incoming edges for each reticulation node in *N*, removing nodes that are not in any path from *ρ*(*N*) to a leaf *ℓ*∈*X*, and contracting degree-two nodes (i.e., nodes of in-degree one and out-degree one). To contract a degree-two node *w* which has two incident edges (*u*,*w*) and (*w*,*v*), we merge the two edges into one edge (*u*,*v*).

A cluster *B*⊂*X* is a *soft cluster* in *N* if there is a tree *T* displayed in *N* such that *B* is a cluster in *T*. A tree node in a network may represent multiple soft clusters, which could be obtained from different trees displayed in the network. We use *S* *C*(*N*) to denote the set of soft clusters in *N*.

Given *B*⊂*X* and a network *T* on *X*, the CCP asks whether *B* is a soft cluster in *N* [10], which is formulated as below:

- **CLUSTER CONTAINMENT**
- **Instance**: A phylogenetic network *N* over a set of taxa *X* and *B*⊂*X*.
- **Question**: Is *B*∈*S* *C*(*N*)?

Let *N*_1_ and *N*_2_ be two networks over the same set of taxa *X*. *The SRF distance* between them is defined to be (|*S* *C*(*N*_1_)∖*S* *C*(*N*_2_)|+|*S* *C*(*N*_2_)*S* *C*(*N*_1_)|)/2 denoted by *d*_*SRF*_(*N*_1_,*N*_2_).

It is worth noting that the SRF distance is not a strict metric, since two distinct networks may represent the same set of soft clusters and hence the SRF distance between them will be zero [10]. Nevertheless, the SRF distance provides a useful measure of network dissimilarity.

### A decomposition theorem

The key to solving the CCP and computing the SRF distance is the decomposition theorem, which was first proposed by Gunawan et al. [16] for reticulation-visible networks and used later for arbitrary networks in [20].

The decomposition theorem says that an arbitrary network *N* can be decomposed into a set of connected tree components which are separated by reticulation nodes. Specifically, there is a tree component *C*_*r*_ for each *r*∈*R*(*N*)∪{*ρ*(*N*)}, which is either {*c*(*r*)} if *r*∈*R*(*N*) and *c*(*r*)∈*R*(*N*), or a subtree induced by all the tree nodes and leaves that are reachable from *r*. A tree component is *trivial* if it contains only one leaf or if it is empty (for a dummy reticulation node).

A node is *visible* on a leaf *ℓ* if it lies on all the paths from *ρ*(*N*) to *ℓ*. If a node *r*∈*R*(*N*)∪{*ρ*(*N*)} is visible on a leaf *ℓ*, its tree component *C*_*r*_ is *visible* on *ℓ* as well. Given two tree components $C_{r'}\phantom {\dot {i}\!}$ and $\phantom {\dot {i}\!}C_{r^{\prime \prime }}$, *r* ^′^ and $\phantom {\dot {i}\!}C_{r^{\prime }}$ are *right below* $\phantom {\dot {i}\!}C_{r''}$ if a parent of *r* ^′^ is in $\phantom {\dot {i}\!}C_{r^{\prime \prime }}$. A tree component is *exposed* if it contains only one leaf or if all the tree components right below it are trivial.

Obviously, *N* contains at least one exposed non-trivial tree component. In addition, an exposed tree component *C*_*r*_ is visible if and only if *C*_*r*_ contains a leaf or if a reticulation node *r* ^′^ exists right below *C*_*r*_ such that all the parents of *r* ^′^ are in *C*_*r*_.

**Fig. 1:**
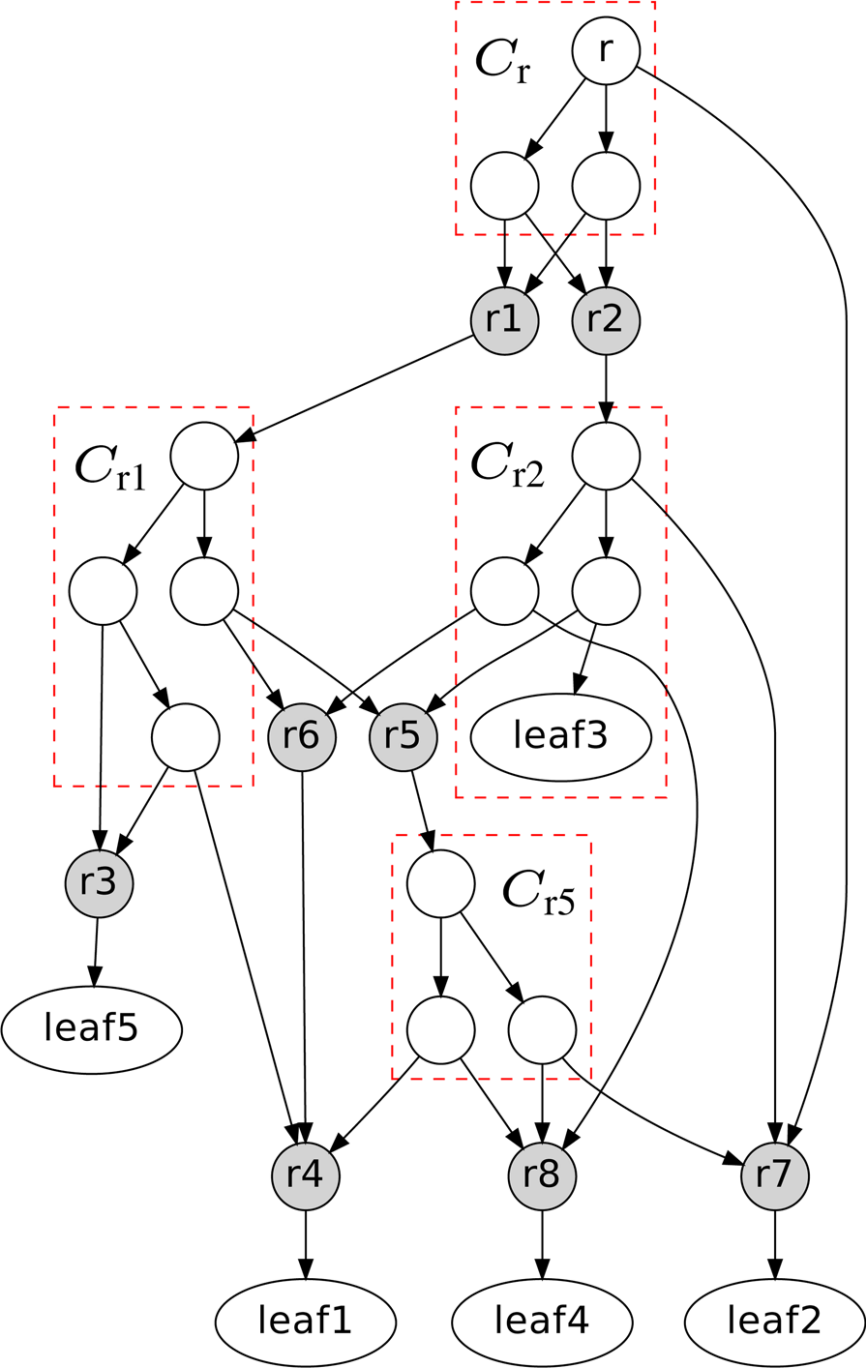
A network *N* and its tree components. There are nine tree components in *N*. Five of these components are non-trivial: *C*_r_, *C*_r1_, *C*_r2_, *C*_r5_, and *C*_r6_, where *C*_r6_={r4}. *C*_r7_ and r7 are right below *C*_r5_, *C*_r2_, and *C*_r_. *C*_r_ is visible on all the leaves. *C*_r1_ and *C*_r2_ are visible, but neither of them is exposed. *C*_r5_ is exposed but not visible

These concepts are briefly illustrated in Fig. 1. See [16, 20] for more details of the decomposition theorem.

### Description of the algorithm

#### The CCP algorithm

With the aid of the generalized decomposition theorem, we extend the linear-time CCP algorithm for reticulation-visible networks in [16] to arbitrary networks.

Roughly speaking, our new CCP algorithm works as follows:

*To determine whether or not a cluster C is in a phylogenetic network N, the algorithm selects a non-trivial exposed component M of N. If M is visible, we either find the negative answer to the problem by working on M or we obtain an instance of the problem that is simpler than the input instance (C,N) in linear time proportional to the size of M. In the latter, we reduce the original instance of the CCP to a simpler instance.*

*If M is not visible, there is then a reticulation node which has a unique leaf child and does not have all parents in M. In this case, two phylogenetic networks* *N*_1_ *and* *N*_2_ *are derived from N, which contain fewer nodes than N. The algorithm is then called on both instances (* *C*,*N*_1_ *) and (* *C*,*N*_2_ *) recursively.*

Although this algorithm seems simple, it has significantly less time complexity when the input network is binary. In the rest of this section, we present a formal description of the algorithm.

Let *N* be a network over *X* and *B*⊂*X*, respectively. We examine a non-trivial exposed tree component *C*_*r*_ of *N*.

The reticulation nodes below *C*_*r*_ are divided into inner-reticulation nodes for which the parents are all in *C*_*r*_, and cross-reticulation nodes for which some parents are not in *C*_*r*_. We use *I* *R*(*C*_*r*_) and *C* *R*(*C*_*r*_) to denote the sets of inner- and cross- reticulation nodes, respectively. For example, in Fig. 1, *I* *R*(*C*_r5_)=*∅* and *C* *R*(*C*_r5_)={r4,r7,r8}.

We use *L*_*r*_ to denote the set of leaves on which *C*_*r*_ is visible:

$$L_{r} = \{c(r') ~|~ r' \in IR(C_{r}) \} \cup L(C_{r}). $$

We use $\check {L}_{r}$ to denote the set of leaves below *C*_*r*_ which are in *B* and on which *C*_*r*_ is not visible:

$$\check{L}_{r} = \{c(r') ~|~ r' \in CR(C_{r}) ~ s.t. ~ c(r') \in B)\}. $$

For example, in Fig. 1, *L*_r5_=*∅* and we can get $\check {L}_{\text {r5}}=\{\text {leaf1}, \text {leaf2}\}$ when assuming *B*={leaf1,leaf2,leaf5}.

Suppose that *L*_*r*_ is non-empty. *C*_*r*_ is then visible with respect to a leaf *ℓ*∈*L*_*r*_. We first check whether *B* is a soft cluster in *C*_*r*_. This can be solved by a linear-time algorithm [16]. If not, we then solve the CCP according to the relationship between *L*_*r*_ and *B*.

Let $\bar {B}=X \backslash B$. If *L*_*r*_∩*B*≠*∅* and $ L_{r} \cap \bar {B} \neq \emptyset $, *B* must be a soft cluster of a node in *C*_*r*_ if *B* is a soft cluster in *N* [16].

If $L_{r} \cap \bar {B} = \emptyset $, *B* may be a soft cluster of *ρ*(*C*_*r*_) or a node in a larger subnetwork containing *C*_*r*_. Assuming that *r* ^′^∈*C* *R*(*C*_*r*_), we then define:

$$\begin{aligned} N_{a}=N-\{(u,r^{\prime}) \in E(N) ~|~ (c(r^{\prime}) \notin B \land u \in V(C_{r}) \}\\ -\{(u,r') \in E(N) ~|~ (c(r^{\prime}) \in B \land u \notin V(C_{r})) \}. \end{aligned}  $$

The leaves below the root of *C*_*r*_ in *N*_*a*_ (i.e., $\phantom {\dot {i}\!}L([\rho (C_{r})]_{N_{a}})$) are then $\phantom {\dot {i}\!}L_{r} \cup \check {L}_{r}$. We denote $\phantom {\dot {i}\!}L([\rho (C_{r})]_{N_{a}})$ as $\hat {B}$ for convenience.

Since *L*_*r*_⊆*B* and $\check {L}_{r} \subseteq B$, $\hat {B} \subseteq B$. If $\hat {B} = B$, *B* is a soft cluster of *ρ*(*C*_*r*_) in *N*_*a*_. Otherwise, if $\hat {B} \subset B$, we set:

1 $$ \left\{ \begin{array}{l} B' = (B \cup \{\ell\}) \backslash \hat{B}, \\ N_{a}'=N-[\rho (C_{r})]_{N_{a}}+\ell. \end{array}  \right.  $$

If *L*_*r*_∩*B*=*∅*, *B* may be a soft cluster of a node in *C*_*r*_ if $\check {L}_{r} \neq \emptyset $. Otherwise, when *B* is not a soft cluster of a node in *C*_*r*_ and *r* ^′^∈*C* *R*(*C*_*r*_), we define:

$$\begin{aligned} N_{b}=N-\{(u,r') \in E(N) ~|~ (c(r') \notin B \land u \notin V(C_{r}))\}\\ -\{(u,r') \in E(N) ~|~ (c(r') \in B \land u \in V(C_{r}))\}. \end{aligned} $$

We can then set:

2 $$ N_{b}' =N-\left[\rho (C_{r})\right]_{N_{b}} + \ell.   $$

With this notation, we can get Theorem 1 for arbitrary networks, which is similar to a theorem proved for reticulation-visible networks in [16]. Theorem 1 is proved in the Additional file 1.

##### **Theorem 1**

Assume that *C*_*r*_ is a non-trivial, exposed and visible tree component in a network *N* over the taxa set *X*, and that *B*⊂*X*. Let *L*_*r*_, $\hat {B}$, *B* ^′^, *N* *a*′, and *N* *b*′ be defined above.

- *(i)* If $\hat {B} \subset B$, *B* is a soft cluster in *N* if and only if *B* ^′^ is a soft cluster in *N* *a*′.
- *(ii)* If *B* is not a soft cluster of a node in *C*_*r*_ and *L*_*r*_∩*B*=*∅*, *B* is a soft cluster in *N* if and only if *B* is a soft cluster in *N* *b*′.

Suppose that *C*_*r*_ is not visible. If *C*_*r*_≠{*c*(*r*)}, there is at least one reticulation node *r* ^′^ right below *C*_*r*_ such that $\phantom {\dot {i}\!}C_{r'}$ is trivial and at least one parent of *r* ^′^ is not in *C*_*r*_. If *C*_*r*_={*c*(*r*)} and $c(r)=r'\phantom {\dot {i}\!}$, then at least one parent of *r* ^′^ is not *r*. We can now define:

3 $${} N' = \begin{array}{ll} N-\{(u, r')\in E(N)~|~ u\not\in C_{r}\} & \text{if}\ C_{r} \neq \{c(r)\}\\ N-\{(u, r')\in E(N)~|~ u \neq r\} & \text{if}\ C_{r} = \{c(r)\}\\ \end{array}   $$

and

4 $${} N^{\prime\prime}= \begin{array}{ll} N-\{(u, r')\in E(N)~|~ u\in C_{r}\} & \text{if}\ C_{r} \neq \{c(r)\}\\ N-\{(u, r')\in E(N)~|~ u = r\}. & \text{if}\ C_{r} = \{c(r)\}\\ \end{array}   $$

Clearly, *B* is a soft cluster in *N* if and only if *B* is a soft cluster in either *N* ^′^ or *N* ^″^.

**Fig. 2:**
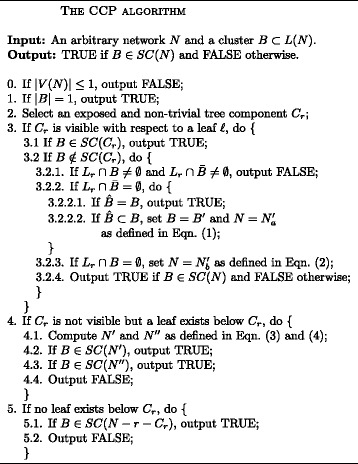
An algorithm for solving the CCP on an arbitrary network

In consideration of all the cases above, we have come up with an algorithm for solving the CCP on an arbitrary network, which is given in Fig. 2.

#### The SRF distance algorithm

We now use the CCP algorithm to compute the SRF distance between two arbitrary networks on the same taxa set *X*.

For *X*, we define a *k-cluster* as a cluster having *k* taxa. We enumerate all the possible clusters over *X* by generating all the *k*-clusters of *X* for each *k* ranging from 1 to |*X*|−1. We then call the CCP algorithm on each cluster to see whether it is a soft cluster in only one network.

The time complexity of this SRF distance algorithm is *O*(2^|*L*(*N*)|^ *T*(*N*)), where *T*(*N*) is the time complexity of the CCP algorithm.

The program for computing the SRF distance in Dendroscope first finds trees displayed in each network, then extracts clusters from these trees to get the soft clusters in each network, and finally traverses the two sets of soft clusters to compute their symmetric difference. If the networks are bi-combining, the time complexity for this method is *O*(2|*L*(*N*)|∗2^|*R*(*N*)|^+2*q*), where *q* is the number of the soft clusters in a network. We will compare this approach and our SRF distance program in next section.

## Results and discussion

### Performance of the CCP program

In this subsection, we first analyze the time complexity of the CCP algorithm. We then report the performance of the CCP program on both simulated and empirical networks. The simulated networks were generated by using a network generator reported by Zhang [17].

#### Theoretical analysis of the time complexity

According to the analysis in [16], the runtime of Step 3 of the CCP algorithm is *O*(|*E*(*C*_*r*_)|), where *E*(*C*_*r*_) is the set of edges in the tree component *C*_*r*_. Thus the time complexity of the CCP algorithm is *O*((*m*+1)|*E*(*N*)|) for a general network *N*, where *m* is the number of times Step 3 is executed. Note that *m* should be an exponential function of |*R*(*N*)| because of the NP-completeness of the CCP. If *N* is a bi-combining reduced network, the time complexity of the CCP algorithm is (2^0.694|*R*(*N*)|^|*E*(*N*)|) [20].

We denote log2(*m*) as *b*(*N*,*B*) and call it the *effective reticulation number* of the CCP algorithm for the network *N* and the cluster *B* [20]. We use *b*(*N*)= max*B* *b*(*N*,*B*) to represent the effective reticulation number of the CCP algorithm for the network *N*.

To the best of our knowledge, the only previously known algorithm for solving the CCP on an arbitrary network is the naive algorithm which considers all the trees displayed in a network and checks whether the input cluster is in one of them. The number of possible trees displayed in a network *N* can be as large as $\prod _{r\in R(N)} deg^{-}(r)$, where *d* *e* *g* ^−^(*r*) is the in-degree of *r*. This number equals 2^|*R*(*N*)|^ when *N* is bi-combining. It takes *O*(|*L*(*T*)|) time to check whether a cluster is in a tree *T* [10]. Thus the effective reticulation number seems to be a good indicator of the efficiency of the CCP algorithm. If log2(*m*) is smaller than |*R*(*N*)|, the CCP algorithm will be faster than the naive algorithm in theory.

#### Performance on random networks

We examined the performance of the CCP program on random networks in term of the effective reticulation number. The tests were done on computers each with 32 GB RAM and a 2.1 GHz AMD Opteron 32-core CPU.

We tested the CCP program on random networks with 10 to 30 leaves and 10 to 80 reticulation nodes. Given that the number of clusters over 15 leaves is huge, it was hard to conduct the evaluation on the whole space of clusters. We therefore generated random clusters for testing on networks with more than 15 leaves. According to the results, the effective reticulation number for each network–cluster pair was frequently smaller than half the number of reticulation nodes in the network.

Here, we report the performance of the CCP program on five groups of networks with 10 leaves and all the possible 1022 (=2^10^−2) clusters. Each group contained 20 networks, and the networks in the *k* ^th^ group had 5(1+*k*) reticulation nodes for each *k* from 1 to 5. The wall clock time on 102,200 (=5×20×1022) network–cluster pairs was 15 minutes and 15 seconds, implying that on average, the program took about one centisecond for each network–cluster pair.

**Fig. 3:**
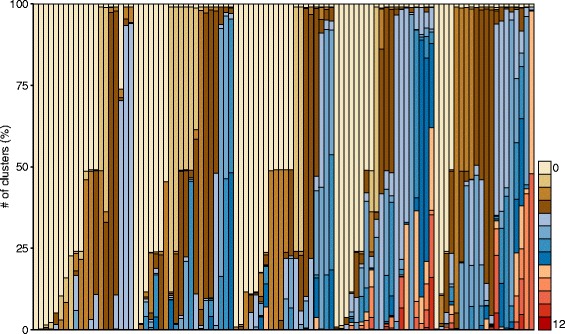
Summary of the performance of the CCP program on five groups of random networks with 10 leaves. Along the *x*-axis, the five groups were arranged from left to right in increasing order of the number of reticulation nodes. The 20 networks in each group were arranged roughly in increasing order of the smallest effective reticulation number. Each stacked bar in a column represents the percentage of clusters that had the same effective reticulation number when the program ran them against the corresponding network

Figure 3 shows the percentages of the clusters in the entire cluster space with the same effective reticulation numbers for each network. Several facts were observed from the test. Firstly, the effective reticulation numbers for the networks in each group increase with the number of reticulation nodes. For example, the effective reticulation numbers for most networks are <5 for the first group, whereas the effective reticulation numbers for more than half of the networks are >9 for the last group. Secondly, there are at least three distinct values of effective reticulation numbers for each network and all the clusters, except for five networks. The effective reticulation number of value one appears for all the networks, since it is easy to determine whether the trivial clusters are soft clusters in a network. Thirdly, the highest effective reticulation number 12 only appears for the 12th network in the last group and one cluster, which is barely seen in Fig. 3 because of the extremely low percentage.

#### Application to a network in the literature

We selected one of the largest networks in the literature to validate the performance of the CCP algorithm. This is a bi-combining network (denoted *A*, Additional file 1: Figure S1) from [21] that has 7 leaves and 32 reticulation nodes. This network is an ancestral recombination graph reconstructed to study the phylogenetic relationships among the M2 double-stranded RNA in the *Rhizoctonia* species complex.

Our test showed that all the clusters on the seven taxa appear as soft clusters in the network *A*. We calculated *b*(*A*,*B*) for each cluster *B* on the seven taxa. The distribution of *b*(*A*,*B*) is shown in Table 1. The effective reticulation number *b*(*A*) is 8, 1/4 of the number of reticulation nodes in *A*. This suggests that the CCP program is about thousands of times as fast as the naive method for this real network.

**Table 1:** The distribution of *b*(*A*,*B*) in the space of clusters over the same set of taxa as the network *A*

*b*(*A*,*B*)		0		2		4		6		8
#Cluster		8		3		45		49		21

#Cluster refers to the number of soft clusters with the same value of *b*(*A*,*B*)

### Performance of the program for the SRF distance on random networks

In this subsection, we first compare the program in Dendroscope and our program for the SRF distance. We then report the performance of a parallel version of our program.

The tests were performed on computers each with 128 GB RAM and a 2.6 GHz Intel Xeon E5-2690 24-core CPU. For the generation of random networks, we considered six cases. In the *k* ^th^ case, we generated six groups of network pairs. The *j* ^th^ group consists of 3000 pairs of networks with 4*k* leaves and *k* *j*/4 reticulation nodes, where *k* was from 1 to 6 and *j*=1,2,4,5,6.

**Fig. 4:**
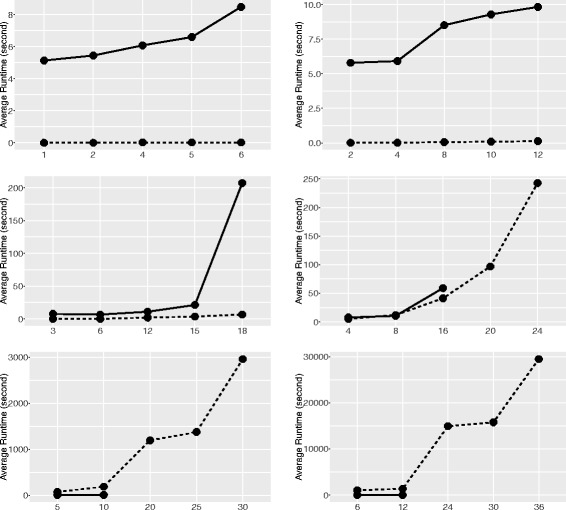
Performance of our program (*dashed line*) and the program in Dendroscope (*solid line*) on random networks. The *x*-axis represents the number of reticulation nodes in a network.The random networks examined had 4 (*top left*), 8 (*top right*), 12 (*middle left*), 16 (*middle right*), 20 (*bottom left*), and 24 leaves (*bottom right*)

For the comparison test, we computed the SRF distance for each pair of networks in every group. The results are summarized in Fig. 4.

Our program ran faster than the program in Dendroscope for networks with up to 16 leaves. However, our program became slower than the latter when there were more than 16 leaves. This is reasonable, since the number of clusters increases exponentially with the number of taxa and it takes even long time for our program to merely list all the possible clusters when there were more than 16 leaves.

Additionally, the memory usage of our program was extremely low compared with the program in Dendroscope. The memory usage of the Dendroscope program increased rapidly with the number of reticulation nodes in a network. For example, the average maximum resident memory for networks with 12 leaves and 18 reticulation nodes was around 95 GB, which is approximately six times that for networks with 12 leaves and 15 reticulation nodes. Because of this, the average runtime of the Dendroscope program for networks with 12 leaves and 18 reticulation nodes sharply increased. During test, the Dendroscope program failed to get results for networks with more than 12 leaves and 20 reticulation nodes. Hence, some data points are missing for the Dendroscope program in the two panels at the bottom in Fig. 4. In contrast, our program can run on networks with more than 30 reticulation nodes. Even for networks with 24 leaves and 36 reticulation nodes, the average maximum resident memory of our program was less than 32 MB. Thus the test shows that our program is computationally efficient when the number of reticulation nodes in the input network is large.

Although our program runs slow for networks with many leaves, it can be easily parallelized for speeding up. We used OpenMP to implement a parallel version of it by parallelizing the enumerations of clusters. This parallel version ran at least 20 times faster than the original program with slightly extra memory. For 3000 pairs of networks each with 20 leaves and 25 reticulation nodes, the parallel version finished in about 36 seconds with less than 40 MB memory on average.

### Computing the SRF distances on real biological data

In this subsection, we examine the SRF distance between phylogenetic networks reconstructed from two datasets in the literature.

#### Computing the SRF distance between networks over a set of grass species

The Proaceae dataset, originally from the Grass Phylogeny Working Group [22], has often been used for validating network reconstruction methods. The dataset contains sequences for six loci: *ITS* (internal transcribed spacer of ribosomal DNA), *ndhF* (NADH dehydrogenase, subunit F), *phyB* (phytochrome B), *rbcL* (ribulose 1,5-biphosphate carboxylase/oxygenase, subunit), *rpoC* (RNA polymerase II, subunit *β* ^″^), and *waxy* (granule bound starch synthase I). Rooted binary gene trees were built for these loci previously by Schmidt [23]. From the six trees, van Iersel et al. [24] constructed 57 subsets of gene trees for comparisons of network reconstruction methods.

A recent method called Hybroscale [25] can compute all the representative networks with the minimum number of reticulation nodes from a set of multiple binary phylogenetic trees. We ran Hybroscale on three subsets of gene trees from the grass dataset, which are on 11, 12, and 15 taxa, respectively (Table 2). The reconstructed networks have less than seven reticulation nodes. Since there are tens of output networks for each input dataset, we computed their pairwise SRF distances to examine their dissimilarity. As shown in Table 2, the average SRF distances between the networks for all the datasets are relatively small, which implies that the computed networks are rather similar.

**Table 2:** The average pairwise SRF distances between the output networks from Hybroscale on three sets of gene trees reported by van Iersel et al. [24]

Gene trees	#Taxa	#Ret	#Networks	Average pairwise
				SRF distance
*rbcL*, *waxy*, *ITS*	11	6	63	12.2
*ndhF*, *rbcL*, *waxy*	12	5	123	8.0
*phyB*, *rbcL*, *rpoC*	15	6	40	1.4

#Ret refers to the number of reticulation nodes in the reconstructed networks

On the other hand, different network reconstruction methods on the same data could produce very different networks. Using five gene trees (*ITS*, *ndhF*, *phyB*, *rbcL*, *rpoC2*), we constructed three networks: a cluster network (Additional file 1: Figure S2) obtained from a program in [26], a galled network (Additional file 1: Figure S3) obtained from a program in [27], and a reticulate network (Additional file 1: Figure S4) obtained from PIRN [28]. Since the original network reconstructed by PIRN had reticulation nodes with more than one child and leaves with more than one parent, it was transformed into an equivalent one satisfying our definition in this paper. The three networks have 18, 7, and 13 reticulation nodes and contain 445, 261, and 209 soft clusters, respectively. The SRF distance between the cluster network and the galled network is 199. The SRF distance between the galled network and the reticulate network is 118. The SRF distance between the reticulate network and the cluster network is 185. This suggests that the galled network is more similar to the reticulate network than to the cluster network. This also reflects that the SRF distance is sensitive to the structural properties of phylogenetic networks.

#### Computing the SRF distance between networks over six mosquito species

To study phylogenetic relationships and introgression among six mosquito species in the *Anopheles gambiae* species complex, Fontaine et al. [5] constructed a network (denoted *M*1) by employing tree-based methods on the whole-genome sequences. Later, Wen et al. [29] rebuilt a similar network (denoted *M*2) for the six species by directly applying a network inference method on the gene trees. The two networks are shown in Additional file 1: Figure S5. *M*1 has three reticulation nodes and *M*2 has four reticulation nodes. There are 18 and 24 soft clusters in *M*1 and *M*2, respectively. The SRF distance between *M*1 and *M*2 is 7, implying that the two networks are still quite different in the embedded soft clusters.

### Comparison of the RF distance and the SRF distance

Although the RF and SRF distances were proposed to measure the dissimilarity of networks, their relationship is unclear [10]. In this subsection, we present our preliminary comparison of these two measures.

**Fig. 5:**
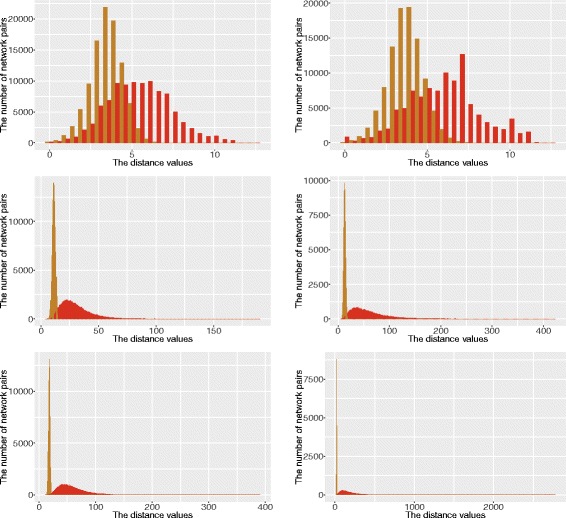
The distribution of the RF (*orange*) and SRF (*red*) distances between random networks. Histograms of the number of network pairs with *k* leaves and *m* reticulation nodes, where (*k*,*m*)=(5,10) (*top left*), (5, 20) (*top right*), (10, 10) (*middle left*), (10, 20) (*middle right*), (15, 10) (*bottom left*), and (15, 20) (*bottom right*)

Given a fixed number of leaves and reticulation nodes, we generated 100,000 random network pairs and computed their RF and SRF distances. Figure 5 shows the distributions of these two measures in the space of networks with different numbers of leaves and reticulation nodes. The results suggest the following three facts:

(i.) There are at least as many soft clusters as clusters in a network. Therefore, as expected, the SRF distance has larger range than the RF distance.
(ii.) The RF distance seems to have a normal distribution of small mean and small variance.
(iii.) The distribution of the SRF distances seems not to be normal. It is skewed towards small distances (especially for networks with more leaves) and a small fraction of network pairs had much larger SRF distances than the average SRF distance.

Taken altogether, these three facts indicate that the SRF distance is a fine metric for networks and hence more suitable than the RF distance for measuring the dissimilarity of networks.

## Conclusions

The generalized decomposition technique developed in [20] was shown to be powerful for solving the TCP on arbitrary networks. In this work, by applying this technique, we have developed efficient algorithms for solving the CCP and computing the SRF distance for arbitrary networks. These two algorithms were implemented in C.

Both programs facilitate reconstructing and validating network models in evolutionary and comparative genomics. Our simulation experiments showed that the SRF distance program ran fast for networks with an intermediate number of leaves and reticulation nodes. Therefore, the SRF distance program is ready for assessing a network reconstructed by a new method via comparing it with other networks.

## Additional file

Additional file 1 Supplementary material. Supplementary material contains the proof for Theorem 1 and Supplementary Figures. (PDF 229 kb)
