# Supplementary material for: A program to compute the soft Robinson–Foulds distance between phylogenetic networks
Source: BMC Genomics. 2017 Mar 14;18(Suppl 2):111. doi: 10.1186/s12864-017-3500-5 (PMC5374702; doi:10.1186/s12864-017-3500-5)
Supplement: Supplementary file 1 — Supplementary material. Supplementary material contains the proof for Theorem 1 and Supplementary Figures. (PDF 229 kb) [file 12864_2017_3500_MOESM1_ESM.pdf]

Supplementary material for "A Program to Compute the Soft Robinson–Foulds Distance between Phylogenetic Networks"

Bingxin Lu[1], Louxin Zhang[2], and Hon Wai Leong[1]

[1]Department of Computer Science, National University of Singapore, Singapore 117417, Singapore
[2]Department of Mathematics, National University of Singapore, Singapore 119076, Singapore

This supplementary material contains the proof for Theorem 1 and Supplementary Figures.

Proof of Theorem 1

(i). Assume that B' is a soft cluster in N'_a. Suppose B' is a soft cluster of a node u. Here, ℓ is a leaf below u. If we re-expand ℓ into $[\rho(C_r)]_{N_a}$ to obtain N, the soft cluster of u will become $(B \backslash \hat{B}) \cup \hat{B}$, namely B. Hence, B is a soft cluster in N.

Assume that B is a soft cluster in N. Suppose B is a soft cluster of a node v in a tree T, where $T = N - E$ and $E \subset E(N)$. Because $\hat{B} \subset B$, B contains a leaf $\bar{\ell}$ which is not below $\rho(C_r)$. As $\bar{\ell}$ is below v in T, v must be above $\rho(C_r)$ in T.

Let $r' \in CR(C_r)$ and $c(r') \in B$. Here, r' has at least one parent in C_r, and $c(r')$ is a leaf below v in T since C_r is exposed. Let $(p_{r'}, r') \in E(N)$ such that $p_{r'} \in C_r$, and $(p'_{r'}, r') \in E$.

Let $T' = T - \{(p'_{r'}, r')\} + \{(p_{r'}, r')\}$. $\hat{B}$ is then the cluster of $\rho(C_r)$ in T' and B is the cluster of v. After replacing $[\rho(C_r)]_{N_a}$ with ℓ to get N'_a, the cluster of v is $(B \cup \ell) \backslash \hat{B}$. Hence, B' is a soft cluster in N'_a.

(ii) Assume that B is a soft cluster in N'_b. Since N'_b is a subnetwork of N, B is a soft cluster in N.

Assume that B is a soft cluster in N. Suppose B is a soft cluster of a node v in a tree T, where $T = N - E$ and $E \subset E(N)$.

Since B is not a soft cluster of a node in C_r, $L_r \cap B = \emptyset$ and $\rho(C_r)$ is visible on leaves in L_r, $\rho(C_r)$ is not below v in T and v is not below $\rho(C_r)$ in T either.

Let $r' \in CR(C_r)$; r' then has at least one parent $p_{r'}$ in C_r.

If $c(r') \in B$, $c(r')$ is a leaf below v in T since C_r is exposed. Then $p'_{r'}$ is not in C_r.

If $c(r') \notin B$, $p'_{r'}$ may or may not be in C_r. Suppose $(p'_{r'}, r') \in E$. If $p'_{r'} \notin C_r$, we can replace $(p'_{r'}, r')$ with $(p_{r'}, r')$ and define $T' = T - \{(p'_{r'}, r')\} + \{(p_{r'}, r')\}$. The cluster of v in T' is then the same as the cluster of v in T. After replacing $[\rho(C_r)]_{N_b}$ with ℓ to get N'_b, the cluster of v is still B. Hence, B is a soft cluster in N'_b.

A phylogenetic network over seven fungi species

Figure S1 shows an ancestral recombination graph over seven fungi species, which was reconstructed to study the phylogenetic relationships among the M2 double-stranded RNA in the Rhizoctonia species complex [1].

Three phylogenetic networks reconstructed from a grass dataset

Figure S2, Figure S3, and Figure S4 show three different kinds of phylogenetic networks, which were reconstructed from five gene trees (*ITS*, *ndhF*, *phyB*, *rbcL*, *rpoC2*) of a grass dataset [2] by three different algorithms [3, 4, 6], respectively.

Two phylogenetic networks reconstructed over six mosquito species

Figure S5 shows two phylogenetic networks reconstructed over six mosquito species, which were reported in [7] and [8], respectively.

References

[1] Charlton, N. D., Carbone, I., Tavantzis, S. M., and Cubeta, M. A. (2008) Phylogenetic relatedness of the M2 double-stranded RNA in Rhizoctonia fungi. *Mycologia*, **100**(4), 555–564.

[2] van Iersel, L., Kelk, S., Rupp, R., and Huson, D. (2010b) Phylogenetic networks do not need to be complex: using fewer reticulations to represent conflicting clusters. *Bioinformatics*, **26**(12), i124–i131.

[3] Huson, D. H. and Rupp, R. (2008) Summarizing multiple gene trees using cluster networks. In *Algorithms in Bioinformatics (WABI)*, Vol. 5251 of *Lecture Notes in Bioinformatics*, Springer, Berlin, pp. 296-305.

[4] Huson, D. H., Rupp, R., Berry, V., Gambette, P., and Paul, C. (2009) Computing galled networks from real data. *Bioinformatics*, **25**(12), i85–i93.

[5] Huson, D. H. and Scornavacca, C. (2012) Dendroscope 3: An interactive tool for rooted phylogenetic trees and networks. *Syst. Biol.*, **61**(6), 1061–1067.

[6] Wu, Y. (2010) Close lower and upper bounds for the minimum reticulate network of multiple phylogenetic trees. *Bioinformatics*, **26**(12), i140–i148.

[7] Fontaine, M. C., Pease, J. B., Steele, A., Waterhouse, R. M., Neafsey, D. E., Sharakhov, I. V., Jiang, X., Hall, A. B., Catteruccia, F., Kakani, E., *et al.* (2015) Extensive introgression in a malaria vector species complex revealed by phylogenomics. *Science*, **347**(6217), 27–28.

[8] Wen, D., Yu, Y., Hahn, M. W., and Nakhleh, L. (2016) Reticulate evolutionary history and extensive introgression in mosquito species revealed by phylogenetic network analysis. *Mol. Ecol.*, **25**(11), 2361–2372.

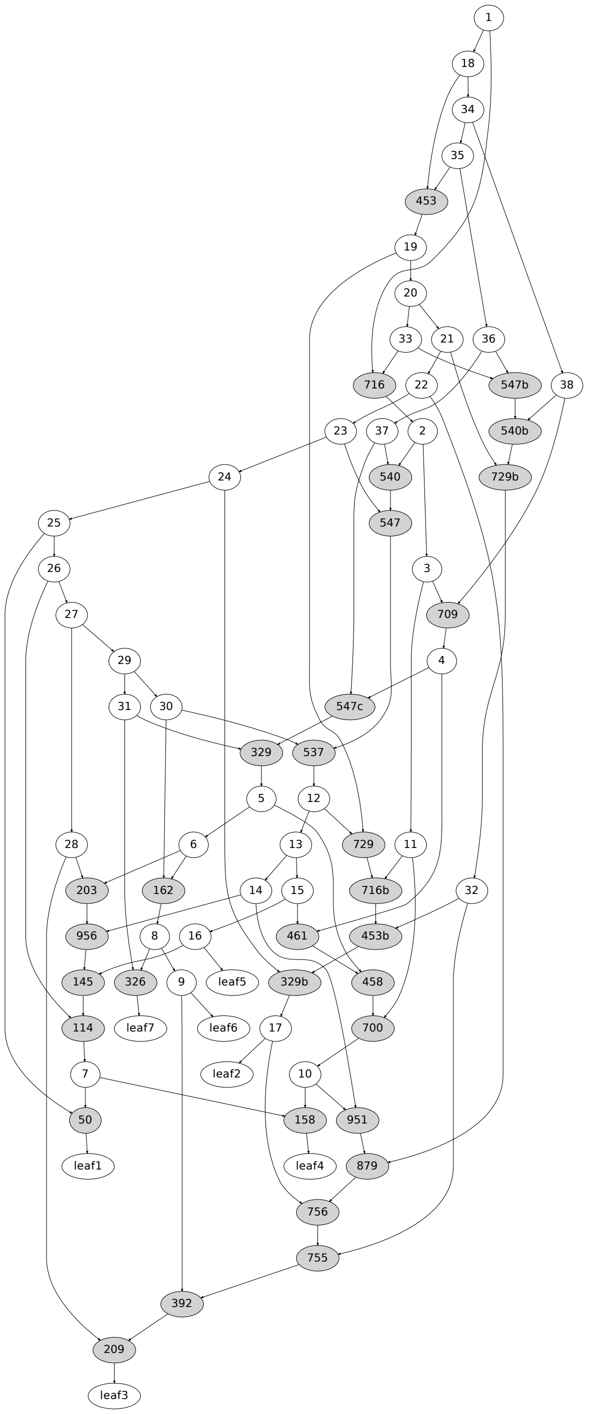

Figure S1: A bi-combining network redrawn from [1].

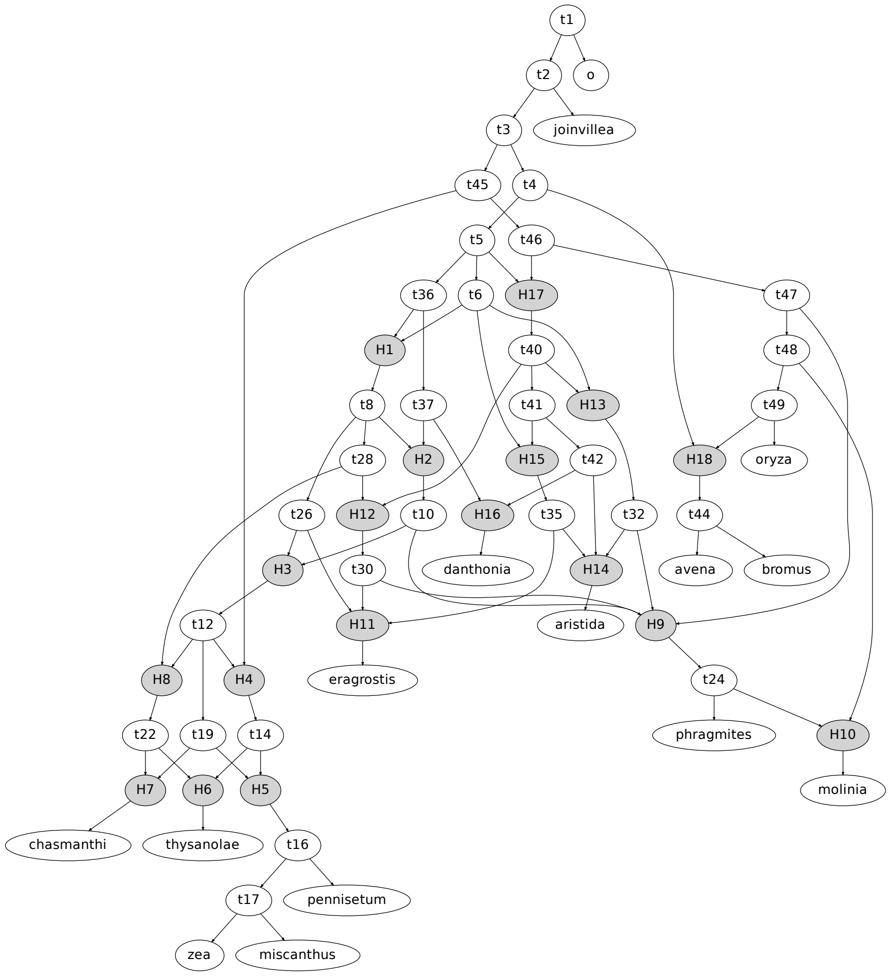

Figure S2: A cluster network reconstructed by Dendroscope [5] from five gene trees of a grass dataset.

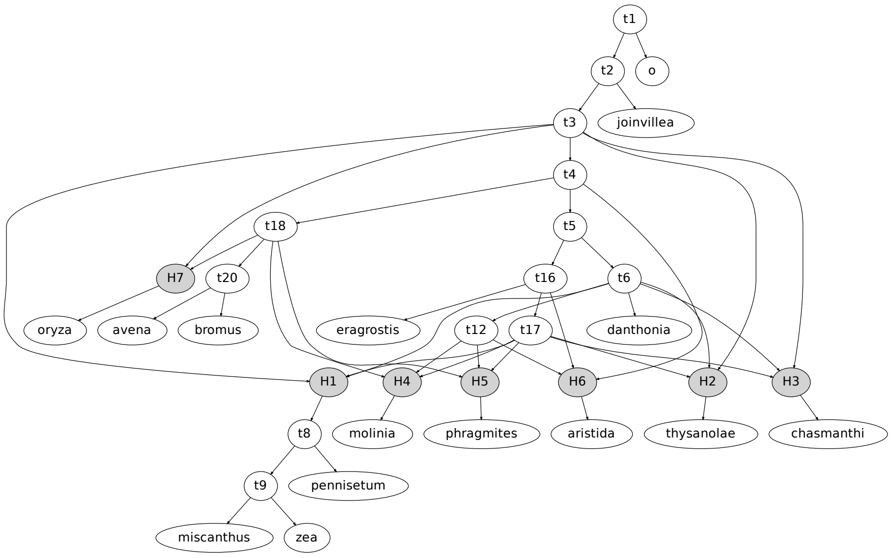

Figure S3: A galled network reconstructed by Dendroscope [5] from five gene trees of a grass dataset.

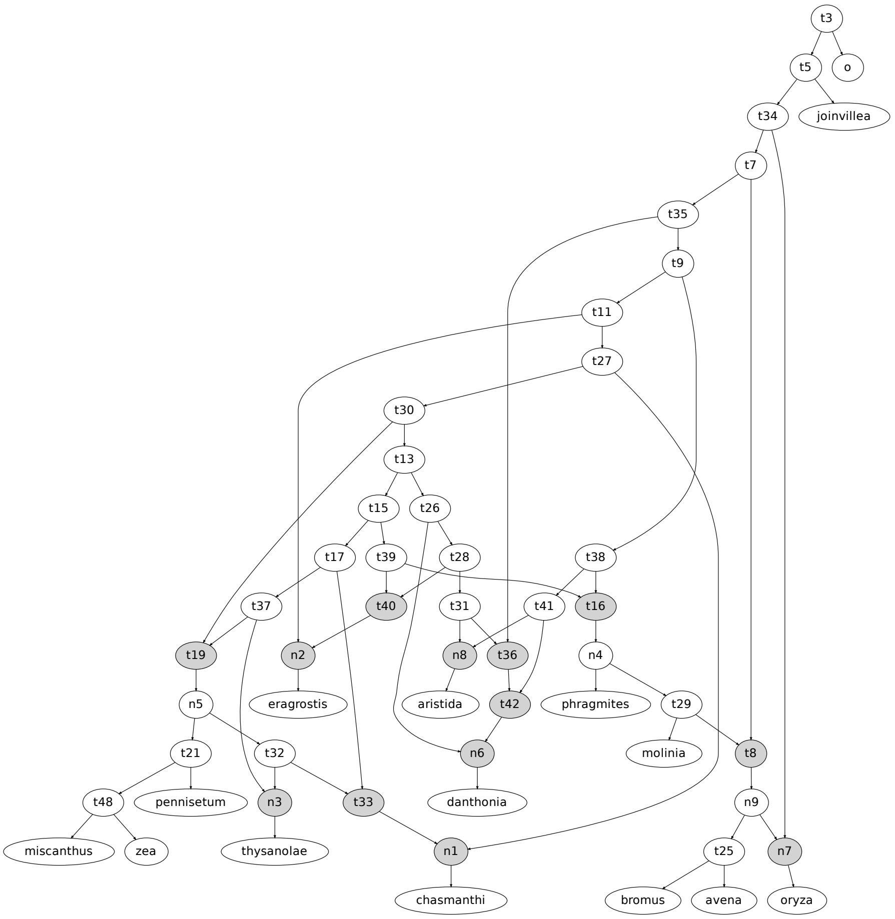

Figure S4: A reticulate network reconstructed by PIRN [6] from five gene trees of a grass dataset.

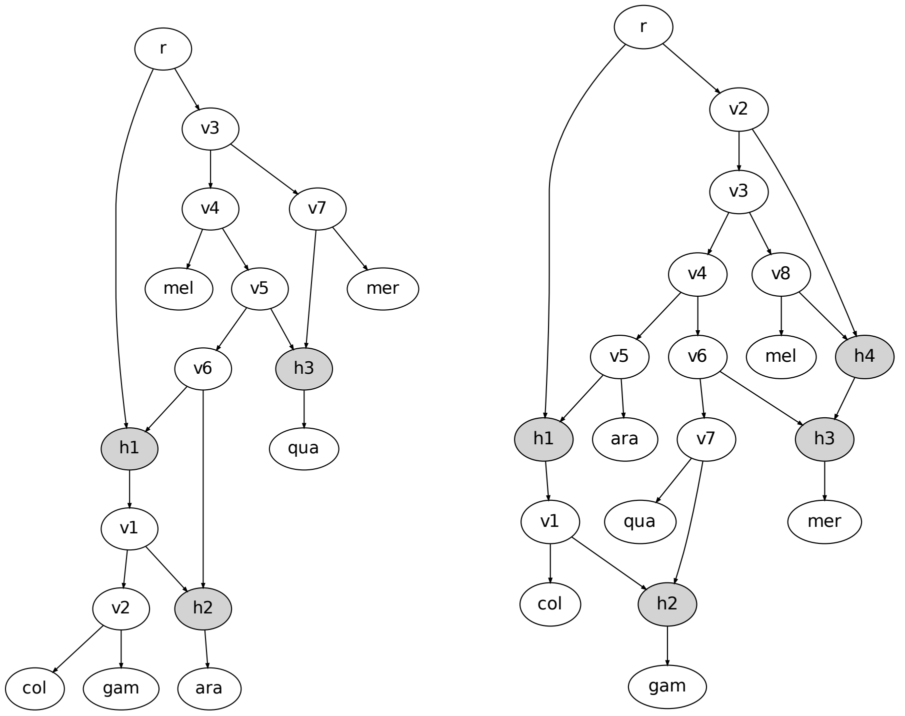

Figure S5: Left panel: A phylogenetic network redrawn from Figure 1C in [7]. Right panel: A phylogenetic network redrawn from Figure 6 in [8].
